# Nutrient status not secondary metabolites drives herbivory and pathogen infestation across differently mycorrhized tree monocultures and mixtures

**DOI:** 10.1016/j.baae.2020.09.009

**Published:** 2021-09

**Authors:** Olga Ferlian, Esther-Marie Lintzel, Helge Bruelheide, Carlos A. Guerra, Heike Heklau, Stephanie Jurburg, Paul Kühn, Ainhoa Martinez-Medina, Sybille B. Unsicker, Nico Eisenhauer, Martin Schädler

**Affiliations:** a German Centre for Integrative Biodiversity Research (iDiv) Halle-Jena-Leipzig, Deutscher Platz 5e, 04103 Leipzig, Germany; b Institute of Biology, Leipzig University, Deutscher Platz 5e, 04103 Leipzig, Germany; c Institute of Biology/Geobotany and Botanical Garden, Martin Luther University Halle-Wittenberg, Am Kirchtor 1, 06108 Halle (Saale), Germany; d Plant-Microorganism Interaction Unit, Institute of Natural Resources and Agrobiology of Salamanca (IRNASA-CSIC), Calle Cordel de Merinas, 40, 37008 Salamanca, Spain; e Department of Biochemistry, Max Planck Institute for Chemical Ecology, Hans-Knöll-Straße 8, 07745 Jena, Germany; f Department of Community Ecology, Helmholtz Centre for Environmental Research – UFZ, Theodor-Lieser-Str. 4, 06120 Halle (Saale), Germany

**Keywords:** Biodiversity-ecosystem functioning, Functional diversity, Leaf elemental concentration, Multitrophic interaction, Mycorrhiza, Plant defence, Plant-insect interaction, Plant-pathogen interaction, Specialised metabolites, Structural equation modelling

## Abstract

Research aimed at understanding the mechanisms underlying the relationship between tree diversity and antagonist infestation is often neglecting resource-use complementarity among plant species. We investigated the effects of tree species identity, species richness, and mycorrhizal type on leaf herbivory and pathogen infestation. We used a tree sapling experiment manipulating the two most common mycorrhizal types, arbuscular mycorrhiza and ectomycorrhiza, via respective tree species in monocultures and two-species mixtures. We visually assessed leaf herbivory and pathogen infestation rates, and measured concentrations of a suite of plant metabolites (amino acids, sugars, and phenolics), leaf elemental concentrations (carbon, nitrogen, and phosphorus), and tree biomass. Tree species and mycorrhizal richness had no significant effect on herbivory and pathogen infestation, whereas species identity and mycorrhizal type had. Damage rates were higher in arbuscular mycorrhizal (AM) than in ectomycorrhizal (EM) trees. Our structural equation model (SEM) indicated that elemental, but not metabolite concentrations, determined herbivory and pathogen infestation, suggesting that the investigated chemical defence strategies may not have been involved in the effects found in our study with tree saplings. Other chemical and physical defence strategies as well as species identity as its determinant may have played a more crucial role in the studied saplings. Furthermore, the SEM indicated a direct positive effect of AM trees on herbivory rates, suggesting that other dominant mechanisms, not considered here, were involved as well. We found differences in the attribution of elemental concentrations between the two rates. This points to the fact that herbivory and pathogen infestation are driven by distinct mechanisms. Our study highlights the importance of biotic contexts for understanding the mechanisms underlying the effects of biodiversity on tree-antagonist interactions.

## Highlights

• Research on tree diversity and antagonists often neglects plant complementarity.
• We studied species richness/mycorrhizal type effects on leaf herbivory/pathogens.
• Mycorrhizal type had different effects on herbivory and pathogen infestation.
• Elemental not metabolite concentrations determined leaf damage.ld.

## Introduction

The positive effects of plant diversity on the functioning of ecosystems are substantially driven by the complementary use of resources. However, the underlying mechanisms of biodiversity-ecosystem functioning (BEF) relationships are still elusive (Cardinale et al., 2012; Eisenhauer et al., 2016; Grossman et al., 2018). Indeed, most of the studies focus on plants and plant-related ecosystem functions, where empirical evidence for resource-use complementarity is inconsistent (Barry et al., 2019). This calls for the consideration of the trophic complexity of ecosystems. It includes interactions of plants with higher trophic levels, such as insect herbivores and pathogens as major determinants of plant fitness (Hines et al., 2015; Weisser & Siemann, 2008). In forest BEF experiments, there have been few studies and inconsistent findings (Grossman et al., 2018; Schuldt et al., 2017), despite the significance of damage caused by insect herbivores (e.g., Franceschi, Krokene, Christiansen & Krekling, 2005) and pathogens (e.g., Graniti, 1998).

Higher tree diversity can affect the fitness of an individual tree species, e.g., through a decrease in leaf herbivory and pathogen infestation rates (Al-Alouni, Brandl, Auge & Schädler, 2014; Hantsch et al., 2014; Jactel & Brockerhoff, 2007). However, numerous studies have also found neutral and even positive effects of biodiversity on herbivory and pathogen infestation rates (Schuldt et al., 2010, 2017; Vehviläinen, Koricheva & Ruohomäki, 2007). The net effect of tree diversity on herbivory can be regarded as the result of opposing, mutually non-exclusive mechanisms. Negative effects can be the result of an increased diversity and efficiency of natural enemies of plant antagonists in more diverse tree stands (Andow, 1991; Jouveau et al., 2020) as well as a consequence of resource dilution especially for specialised antagonists (resource concentration hypothesis; Barbosa et al., 2009; Castagneyrol, Giffard, Péré & Jactel, 2013; Root, 1973). Positive effects of plant diversity on insect herbivore performance may be driven by positive effects on generalists, in particular through diet mixing for example, (Unsicker, Oswald, Köhler & Weisser, 2008) or herbivore spill-over from preferred to other plants (e.g., Jactel & Brockerhoff 2007; White & Whitham 2000). In the same line, some pathogens depend on the presence of multiple plant species to complete their life cycle (Nguyen et al., 2016). Whilst these hypotheses focus on antagonist-associated processes, there is an increasing recognition of host-associated processes as underlying mechanisms of plant diversity effects on plant antagonists. The complementary use of resources in more diverse plant communities (Loreau & Hector 2001; Oelmann et al., 2007), may lead to higher leaf quality and, consequently, higher rates of herbivory. In contrast, the enhanced resource supply may also be invested in defence strategies and reduce the palatability of plant material for herbivores (Kostenko, Mulder, Courbois & Bezemer, 2017; Mraja, Unsicker, Reichelt, Gershenzon & Roscher, 2011).

The elemental composition of plants determines the plant biomass consumed by herbivores (Cebrian & Lartigue 2004; Sterner, Elser, Fee, Guildford & Chrzanowski, 1997). A better nutrient supply of plants, e.g., within a diverse plant community, can lead to an elevated fitness of the antagonists. At the same time, it may strengthen the community's ability to defend itself against the antagonists. As these mechanisms act antagonistically, predictions on the effects of plant diversity on leaf herbivory and pathogen infestation remain difficult. Furthermore, allocation of elements to plant defence may depend on the probability of antagonist attack and the general benefit of defence (Cipollini, Walters & Voelckel, 2018; Stamp, 2003). To ultimately unravel the underlying relationship between plant diversity, leaf herbivory, and pathogen infestation rates, it is thus crucial to concomitantly study plant elemental concentrations and the main compounds involved in plant defence against herbivores and pathogens.

Mycorrhizal fungi play critical roles in the competitive capabilities of plants (Smith & Read 2010; van der Heijden, Martin, Selosse & Sanders, 2015). The two most common mycorrhizal types are arbuscular mycorrhiza (AM) and ectomycorrhiza (EM). They have distinct foraging strategies and life styles as well as different mechanisms for resource exchange (Bonfante & Genre 2010; Soudzilovskaia et al., 2019). AM fungi completely depend on their host as the sole carbon supplier and, in turn, provide the plant host with soil phosphorus that is often limiting to the plant (Brundrett, 2009; Smith & Read 2010). EM fungi can be obligate or also have a saprotrophic phase, taking up organic and mineral plant resources from various substrates for nutrient exchange with the host. Due to the large hyphae system, EM fungi scavenge more effectively and at further distances from the host roots compared with AM fungi. The mutual interaction between mycorrhizal fungi and plants may lead to an increased nutritional value of leaves and synthesis of defence-related compounds (Fernández et al., 2014; Kaling et al., 2018). Moreover, it has been found that, in the initial phase of mycorrhizal fungal colonisation, plants recognise fungi as invaders triggering similar responses like pathogens (Ferlian et al., 2018a). In this way, mycorrhizal fungi are able to enhance plant immunity by increasing the levels of defence-related metabolites and foster defence-priming (Gange & West 1994; Kempel, Schmidt, Brandl & Schädler, 2010; Martinez-Medina et al., 2016).

The functional diversity of plants in terms of mycorrhizal association may potentially increase resource partitioning amongst plant species (Klironomos, McCune, Hart & Neville, 2000; Wagg, Barendregt, Jansa & van der Heijden, 2015); and mycorrhizal fungi have been proposed to play a critical role in positive BEF relationships (Ferlian et al., 2018a, 2018b). Thus, the effects of plant diversity on antagonists may be co-determined by the effects of mycorrhizal diversity via changes in resource acquisition, plant defences, and the nutritive value of plant tissue.

We investigated the effects of tree species identity and richness as well as mycorrhizal type on leaf herbivory and pathogen infestation rates. We used a tree sapling diversity experiment that manipulates the two most common mycorrhizal types (AM and EM; via suitable tree species selection) and tree species richness (monocultures and two-species mixtures). We measured the concentrations of defence-related plant metabolites (a set of sugars, amino acids, and phenolics) to shed light on the underlying mechanisms between plant diversity and leaf damage. Furthermore, we measured leaf elemental (carbon [C], nitrogen [N], and phosphorus [P]) concentrations reflecting general plant nutrient uptake and leaf palatability. (1) We hypothesised that herbivory and pathogen infestation rates are lower in the tree species mixtures compared to monocultures. Similarly, herbivory and pathogen infestation rates are lower in tree communities being associated with both mycorrhizal types compared to communities with only one dominant mycorrhizal type. This is attributable to the resource concentration hypothesis and the higher defence ability due to partitioning and better exploitation of resources. (2) We further hypothesised that concentrations of defence-related plant metabolites explain a higher proportion of the effects on herbivory and pathogen infestation rates than element concentrations. (3) Due to the different life strategies and specialisation of leaf herbivores and pathogens, the mechanisms behind the effects of plant diversity differ between leaf herbivory and pathogen infestation rates.

## Materials and methods

### Study site

The site is located in Southeastern Germany, at the Bad Lauchstädt Experimental Research Station of the Helmholtz Centre for Environmental Research-UFZ (51°23′ N, 11°53′ E), 115 m a.s.l.; the climate is continental with an annual mean temperature of 8.8 °C and 484 mm mean annual precipitation. The site has silt over calcareous silt as parent rock, and the soil type is Haplic Chernozem developed from Loess with a pH range between 6.6 and 7.4 (Altermann et al., 2005; Ferlian et al., 2018b). For further site characteristics, see Ferlian et al. (2018b).

### Experimental design

In March 2015, we set up tree monocultures and two-species mixtures (‘tree species richness treatments‘) within the framework of the tree diversity experiment MyDiv (Ferlian et al., 2018b). In addition, a mycorrhizal treatment with three levels, tree communities predominantly associated with AM fungi, tree communities predominantly associated with EM fungi, and tree communities with both mycorrhizal types, was established. Tree species naturally associated with either one of the two mycorrhizal types (Wang & Qiu 2006) were planted. This resulted in nestedness of tree species identity in mycorrhizal type identity. As we did not control for mycorrhizal fungal colonisation directly, we use the terms ‘dominance of AM’ and ‘dominance of EM’ for the two treatment levels hereafter.

For the AM-species pool, the following five species were selected: *Acer pseudoplatanus, Aesculus hippocastanum, Fraxinus excelsior, Prunus avium*, and *Sorbus aucuparia*. For the EM-species pool, the following five species were selected: *Betula pendula, Carpinus betulus, Fagus sylvatica, Quercus petraea*, and *Tilia platyphyllos*. Per species, we set up two monoculture replicates, ten replicates in two-species mixtures each for AM tree species and for EM tree species, plus 25 replicates in two-species mixtures, in which there was always an AM tree species combined with an EM tree species. Thus, all possible species combinations were implemented. Specific species compositions were not replicated to not confound species diversity effects with that of community composition. Overall, 65 plots were set up in a random spatial arrangement. Each plot contained four two- to three-year-old tree individuals planted in a rectangular pattern. Distance between trees was 15 cm and between plots 1 m. All plots were covered with a water-permeable polypropylene tarp to minimise competition and interference with weeds.

To validate the mycorrhizal type treatment in our study, we assessed the colonisation rates with AM and EM morphologically in all ten tree species as described in Appendix A: Methods A.1. We found trends of differing mycorrhizal colonisation rates between AM and EM trees; and variation was overall relatively high (e.g., mean frequency ratio in AM trees: 4.43 ± 4.58; mean frequency ratio in EM trees: 3.30 ± 3.72; Appendix A: Fig. A.1).

### Leaf damage

At the beginning of September 2016 (after a duration of 18 months), 15 sun leaves per tree individual were randomly selected from different branches. Visual signs of leaf damage due to insect herbivore and pathogen attack were recorded. In particular, we differentiated between four herbivore categories (chewer, hole-feeder, miner, and sucker) and two fungal pathogen categories (rust and mildew; hereafter called feeding type). However, damage by sucking insects could not be added to the analyses as rates were too low. Percentage of herbivory and pathogen infestation was calculated from presence/absence data within these fifteen leaves within each category and in total representing the respective tree individual. Means of leaf damage were calculated from these values per species and plot.

### Leaf elemental concentrations

Right after leaf damage assessment, five intact sun leaves (without signs of herbivory or pathogen infestation) per tree individual were randomly collected from different branches, pooled to one sample per tree species within a plot, and cooled. Leaves were frozen at −80 °C until processing. A subsample of leaves was dried at 60 °C and ground to fine homogeneous powder with a ball mill (MM 400, Retsch, Haan, Germany). Five mg of leaf material were transferred into tin capsules. Leaf total C and total N concentrations were measured with an elemental analyser (vario EL cube; Elementar Analysensysteme GmbH, Hanau, Germany). For measurements of total leaf P concentrations, a further aliquot of 500 mg powder was dissolved using microwave digestion and 5 ml HNO_3_ and 0.5 ml H_2_O_2_ (200 °C for 30 min, Multiwave, Anton Paar GmbH, Graz, Austria). Samples were measured with an inductively coupled plasma optical emission spectrometer (wave length: 177.5 nm; limit of determination: 0.13 mg/L; Arcos, Spectro Analytical Instruments GmbH, Kleve, Germany). Means of elemental concentrations were calculated per species and plot.

### Leaf chemical compounds

A further subset of leaves was taken from all samples, lyophilised, and ground with a ball mill to fine powder. Ten mg of this leaf powder were extracted with methanol (0.1 ml per mg) containing 0.8 mg/ml phenyl-β-glucopyranoside, 10 µg/ml trifluoromethyl cinnamic acid and 1 mg/ml syringic acid (Sigma Aldrich, St. Louis, USA) as internal standards. The samples were shaken twice with a paint shaker for 30 s (Scandex, Pforzheim, Germany) and then centrifuged for 2 min at 3200 rpm. The supernatants were then used to analyse sugars, amino acids, and phenolics as described in detail in Appendix A: Methods A.2. Briefly, the compounds were measured with an HPLC coupled to a triple‐quadrupole mass spectrometer after diluting the extracts 1:10 with water (in case of sugars and amino acids). In case of amino acids, a 10 µg ml^−1^ mixture of ^15^N/^13^C labelled amino acids (Isotec, Miamisburg, OH, USA) was added to the samples and compound separation was achieved on a Zorbax Eclipse XDB-C18 column (50 × 4.6 mm x 1.8 µm; Agilent, Santa Clara, CA, USA). This column was also used to separate phenolic compounds (Appendix A: Table A.1). Sugars were separated on a hydrophilic interaction liquid chromatography (HILIC) column (15 cm x 4.6 mm x 5 µm, apHera-NH2 Polymer; Supelco, Bellefonte, PA, USA). Means were calculated per species and plot.

### Tree biomass

All trees were harvested in September 2016. Stems were cut at the base to separate aboveground from belowground biomass. Tree individuals were separated. Tree aboveground parts including all woody parts and leaves were weighed per tree individual after drying at 60 °C for one week.

### Statistical analysis

Tree biomass, leaf elemental and compound concentration data were log-transformed which improved homoscedasticity and normal distribution. Data on herbivory and pathogen infestation rates were logit-transformed. The variables tree species richness and mycorrhizal type were merged into one composite variable with the following five levels: monoculture-AM, monoculture-EM, mixture-AM, mixture-EM, and mixture-both mycorrhizal types. Using linear mixed effects models, the effects of the composite variable, elemental concentrations (C and N), and metabolite concentrations (see Appendix A: Table A.2 for a list of compounds) on (total and feeding type-specific) leaf damage were tested. We used random intercept models and tree species identity as random factor. We further used marginal R^2^ and conditional R^2^ to display the proportion of variance explained by the fixed factor alone and by both the fixed and the random factor, respectively. The models using leaf damage and element concentrations were run for all mycorrhizal types separately. Tree species richness was used as a second random factor. Similarly, we conducted linear mixed effects analyses on herbivory and pathogen infestation rates using tree species identity as fixed factor and tree species richness as random factor. We used Tukey's Honestly Significant Difference (HSD) test for pairwise comparisons. Additionally, we grouped compounds into benzyl alcohol derivatives, coumarins, flavan-3-ols, flavone glucosides, and phenolic acids and summed the concentrations of individual compounds in these groups, respectively. Correlations between plant metabolite group concentrations and leaf damage were also tested using a linear mixed effects model. The variables tree species identity and tree species richness were used as random factors.

We, further, performed a redundancy analysis (RDA) and variation partitioning using the metabolite data, which was first transformed into relative values per metabolite. With the variation partitioning, we were able to identify the amount of variance in metabolite profiles explained by tree species identity relative to the variance explained by the other predictor variables. The significance of the RDA, the different fractions in the variance partition analyses and the RDA axes were tested by global permutations, using 999 iterations. Building on this, we conducted a partial RDA (pRDA), to partial out the effects of tree species identity. We used the coordinates of the first two axes of this ordination to look at how the metabolite profiles differed between samples once the effect of tree species was factored out. Analyses were conducted with R (R Development Core Team, 2014) using the packages ‘ggplot2’ (Wickham, 2016), ‘lme4’ (Bates, Mächler, Bolker & Walker, 2015), ‘MuMIn’ (Barton, 2013), and ‘vegan’ (Oksanen et al., 2013).

To disentangle direct from indirect effects of tree species richness and mycorrhizal type on herbivory and pathogen infestation rates in a single model, structural equation models (SEMs) were applied (Grace, 2006). The following variables were scaled and used to set up the model: mycorrhizal type (as binary coded [0, 1] variables AM and EM), tree species richness, leaf P, leaf N, leaf C, tree aboveground biomass, herbivory rates, pathogen infestation rates, and the scores of the first two pRDA axes. The variables AM, EM, and tree species richness represented exogenous variables, whereas the others were treated as endogenous variables. An initial meta-model was created and properly justified based on expert knowledge (see Fig. 3A for a full justification and related hypotheses for all path groups). Using IBM AMOS v21, we conducted an overall model fitting. Model selection was based on a stepwise approach using CAIC values starting with an initial valid model, following the removal of the weakest insignificant path from the model (checking for the decrease of the CAIC). The procedure was repeated until the difference in CAIC between the former and present model was smaller than 2. The resulting SEM was used here as the basis for our results.

## Results

### Effects of tree species identity, richness, and mycorrhizal type on leaf damage

**Fig. 1:**
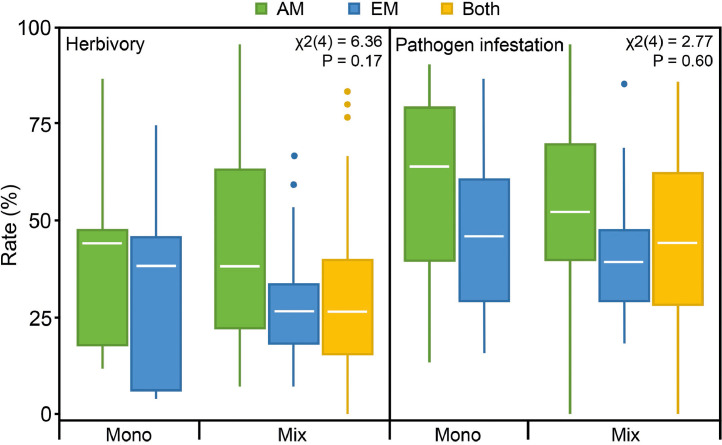
Total herbivory and pathogen infestation rates in tree species monocultures (Mono) and mixtures (Mix) of arbuscular mycorrhizal (AM) communities, ectomycorrhizal (EM) communities and communities with both mycorrhizal types (Both).

Total herbivory rates did not differ significantly between the five levels of the composite treatment variable (Fig. 1) as indicated by linear mixed effects models (β_mono-EM_ = −0.05, SE = 0.52; β_mix-AM_ = 0.16, SE = 0.39; β_mix-EM_ = 0.09, SE = 0.47; β_mix-Both_ = −0.40, SE = 0.37; χ^2^(4) = 6.4; *P* = 0.17). However, there was a trend of lower total herbivory rates in EM compared to AM communities in both monocultures and mixtures. Total herbivory rates tended additionally to be generally lower in mixtures compared to monocultures. Proportions of variation explained by both fixed and random factors (composite treatment and tree species identity, R^2^_m_) and that explained by only the fixed factor (composite treatment, R^2^_c_) were 0.02 and 0.41. Herbivory rates differed highly significantly among tree species in all communities (AM, EM, and Both; Table 1). In AM communities, *S. aucuparia* and *A. hippocastanum* showed the lowest and highest rates, respectively; in EM communities, *F. sylvatica* and *T. platyphyllos* showed the lowest and highest rates, respectively; in communities with both mycorrhizal types, *F. sylvatica* and *A. hippocastanum* showed the lowest and highest rates, respectively.

**Table 1:** Summary of linear mixed effects analyses on total herbivory and pathogen infestation rates as affected by tree species identity, and respective means (± SD). Analyses were conducted separately for arbuscular mycorrhizal (AM) communities (df = 4), ectomycorrhizal (EM) communities (df = 4), and communities with both mycorrhizal types (df = 9). Significant effects (P < 0.05) are given in bold. Letters indicate pairwise differences according to Tukey's Honestly Significant Difference (HSD) test.

	Herbivory			Pathogen infestation		
	χ^2^	P	Mean ± SD	χ^2^	P	Mean ± SD
AM	**56.27**	**< 0.001**		**27.45**	**< 0.001**	
*A. pseudoplatanus*			47.51 ± 10.10^a^			68.35 ± 18.92^a^
*A. hippocastanum*			79.68 ± 5.33^b^			16.35 ± 10.03^b^
*F. excelsior*			22.51 ± 7.44^c^			60.01 ± 14.91^a^
*P. avium*			50.57 ± 16.43^a^			77.51 ± 14.83^a^
*S. aucuparia*			13.63 ± 5.42^c^			51.13 ± 20.08^a^
EM	**25.99**	**< 0.001**		7.52	0.11	
*B. pendula*			31.81 ± 7.67^ab^			30.76 ± 10.78
*C. betulus*			16.40 ± 12.23^ac^			48.35 ± 25.26
*F. sylvatica*			10.85 ± 6.48^c^			37.79 ± 7.73
*Q. petraea*			42.96 ± 13.70^b^			50.58 ± 24.61
*T. platyphyllos*			47.51 ± 22.36^b^			59.46 ± 22.68
Both	**40.42**	**< 0.001**		**31.5**	**< 0.001**	
*A. pseudoplatanus*			31.10 ± 25.12^abc^			59.12 ± 18.47^ab^
*A. hippocastanum*			60.01 ± 26.95^c^			31.68 ± 27.56^a^
*F. excelsior*			28.01 ± 9.01^abc^			36.01 ± 13.83^ab^
*P. avium*			40.68 ± 16.57^bc^			71.35 ± 12.83^b^
*S. aucuparia*			12.01 ± 6.92^ab^			66.68 ± 7.46^ab^
*B. pendula*			31.35 ± 7.31^abc^			32.01 ± 11.46^ab^
*C. betulus*			11.10 ± 5.26^ab^			50.92 ± 29.73^ab^
*F. sylvatica*			9.18 ± 5.70^a^			25.01 ± 12.91^a^
*Q. petraea*			29.80 ± 13.89^abc^			57.65 ± 9.12^ab^
*T. platyphyllos*			50.68 ± 21.66^c^			41.35 ± 12.16^ab^

Similarly, total pathogen infestation rates did not differ significantly between the five treatment levels (β_mono-EM_ = −0.33, SE = 0.69; β_mix-AM_ = −0.66, SE = 0.52; β_mix-EM_ = −0.56, SE = 0.62; β_mix-Both_ = −0.71, SE = 0.50; χ^2^(4) = 2.8, *P* = 0.60; Fig. 1). Like herbivory rates, pathogen infestation rates were lower in EM communities than in AM communities, and mixtures had generally lower rates. Within the mixtures, total pathogen infestation rates of tree communities with both mycorrhizal types were in between that of only AM and only EM communities. Pathogen infestation rates differed significantly among tree species in AM and communities with both mycorrhizal types but not in EM communities (Table 1). In AM communities, *A. hippocastanum* and *P. avium* showed the lowest and highest rates, respectively; in EM communities, *B. pendula* and *T. platyphyllos* showed the lowest and highest rates, respectively; in communities with both mycorrhizal types, *F. sylvatica* and *P. avium* showed the lowest and highest rates, respectively.

### Correlations between leaf elemental concentrations and leaf damage

N concentrations and herbivore mining rates were significantly positively correlated, whereas all other correlations between elemental concentrations and herbivory-related variables were not significant (Table 2).

**Table 2:** Summary of linear mixed effects analyses of regressions between leaf carbon and nitrogen concentrations and total and feeding type-specific herbivory and pathogen infestation rates (fixed factor). Analyses were conducted separately for arbuscular mycorrhizal (AM) communities, ectomycorrhizal (EM) communities, and communities with both mycorrhizal types. Estimates (β) represent the change. Marginal (only fixed effects, R^2^_m_) and conditional (fixed and random effects, R^2^_c_) R^2^-values are given. Significant effects are given in bold (P < 0.05; df = 1).

		AM					EM					Both				
		β	SE	χ^2^	R^2^_m_	R^2^_c_	β	SE	χ^2^	R^2^_m_	R^2^_c_	β	SE	χ^2^	R^2^_m_	R^2^_c_
Total herbivory	Leaf C	−1.90	12.07	0.01	< 0.01	0.87	6.62	10.87	0.30	0.01	0.60	5.50	13.89	0.15	0.01	0.54
Chewer		43.68	31.56	0.99	0.06	0.82	−10.52	18.16	0.30	0.01	0.47	7.96	33.49	< 0.01	< 0.01	0.34
Hole-feeder		−58.20	43.27	2.15	0.10	0.34	−10.87	30.90	0.20	< 0.01	0.04	−6.02	26.18	0.06	< 0.01	< 0.01
Miner		11.78	47.95	0.14	< 0.01	0.80	17.26	30.21	0.09	0.01	0.46	5.34	43.12	0.07	< 0.01	0.53
Total pathogens		−18.50	18.54	1.22	0.06	0.59	**−25.95**	**8.15**	**8.95**	**0.27**	**0.27**	−3.38	12.24	0.14	< 0.01	0.40
Rust		−58.42	38.82	2.48	0.10	0.73	**−20.54**	**8.43**	**5.07**	**0.18**	**0.18**	−4.57	32.21	0.04	< 0.01	0.52
Mildew		−4.73	42.74	0.03	< 0.01	0.49	−9.86	26.33	0.20	< 0.01	0.63	4.40	33.82	0.02	< 0.01	0.79
Total herbivory	Leaf N	0.68	2.09	0.14	< 0.01	0.85	4.64	3.46	1.86	0.03	0.58	2.06	2.76	0.62	0.01	0.49
Chewer		−5.41	5.83	1.00	0.02	0.65	5.18	6.25	0.75	0.02	0.40	10.29	7.28	2.10	0.05	0.30
Hole-feeder		−12.07	10.27	1.51	0.05	0.32	6.78	12.53	0.31	0.01	0.01	4.64	7.40	0.41	0.01	0.01
Miner		**18.25**	**8.23**	**4.14**	**0.07**	**0.79**	10.06	10.27	0.98	0.03	0.38	3.98	8.57	0.26	< 0.01	0.51
Total pathogens		**−7.18**	**3.46**	**4.37**	**0.10**	**0.61**	**−10.69**	**3.48**	**8.67**	**0.25**	**0.34**	−2.97	2.60	1.39	0.03	0.36
Rust		−12.83	7.07	3.43	0.06	0.72	**−10.40**	**3.31**	**8.76**	**0.25**	**0.42**	−1.48	6.46	0.06	< 0.01	0.51
Mildew		−5.13	8.76	0.13	0.01	0.51	−3.71	8.50	0.21	< 0.01	0.63	−0.19	5.84	< 0.01	< 0.01	0.78

C and N concentrations were significantly negatively correlated with total pathogen and rust infestation rates in EM communities (Table 2). Leaf N was negatively correlated with total pathogen rates in AM communities, whereas there were no other significant correlations. In contrast to herbivory rates, all pathogen-related infestation rates were negatively correlated with C and N concentrations (Table 2).

In general, differences between proportions of variation explained by both fixed and random factors (elemental concentration and tree species identity and richness, respectively; Table 2) and those explained by only the fixed factor (elemental concentration) were larger in AM communities compared to EM communities and communities with both mycorrhizal types. Correlations and trends for C concentrations resembled that of N concentrations.

### Effects of tree species richness and mycorrhizal type on plant metabolites

**Fig. 2:**
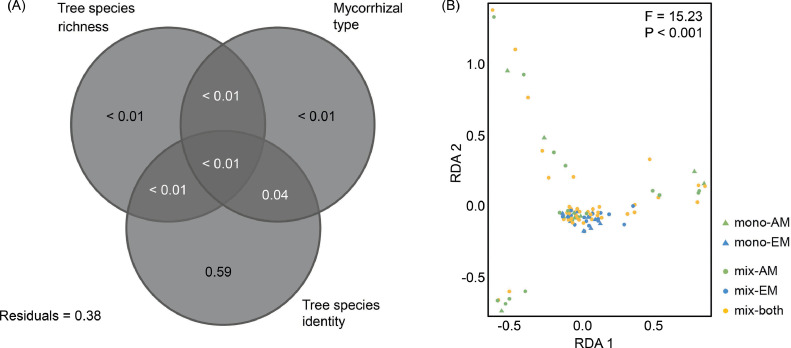
Results of the multivariate analyses of leaf metabolite profiles as affected by tree species identity and richness as well as mycorrhizal type. (A) Venn diagram depicting variation portioning in metabolite concentrations among the three predictor variables and shared variation. (B) Ordination biplot of the redundancy analysis illustrating the relationship between metabolite profiles and tree species richness and mycorrhizal type (as composite variable). mono: monoculture, mix: mixture, AM: arbuscular mycorrhiza, EM: ectomycorrhiza, both: both mycorrhizal types.

The variation partitioning revealed that tree species identity significantly explained most of the variance in the metabolite profile (R^2^_adjusted_ = 0.59, *P* < 0.001; Fig. 2A). Tree species richness and mycorrhizal type explained little variance (R^2^_adjusted_ < 0.01 for both). All of the variance explained by mycorrhizal type was shared with tree species identity (R^2^_adjusted_ = 0.04), and the variance explained exclusively by mycorrhizal type was not significant (*F* = 0.74, *P* = 0.80). The RDA was significant (*F* = 15.23, *P* < 0.001, Fig. 2B) with the first two axes being significant as well (first axis: *F* = 45.63, *P* < 0.001; second axis: *F* = 44.06, *P* < 0.001). Metabolite profiles did not differ significantly between monocultures and mixtures (*F* = 0.52, *P* = 0.86), but differed significantly between tree species (*F* = 18.60, *P* < 0.001) and mycorrhizal types (*F* = 7.43, *P* < 0.001). Subsequently, pRDA was conducted to partial out the effect of tree species identity. The pRDA was not significant (*F* = 0.67, *P* = 0.92, Appendix A: Fig. A.2). The first two axes of the pRDA showed no significant differences between the metabolite profiles of the treatments (F_3,94_ = 0.01, *P* = 0.71). However, metabolite profiles tended to differ between monocultures and mixtures.

### Relations between plant metabolites and leaf damage

Correlations between plant metabolite groups and leaf damage depended on the metabolite group, feeding type, and mycorrhizal type (Appendix A: Table A.3). Benzyl alcohol derivative concentrations were significantly negatively related to herbivore mining (β = - 0.05, SE = 0.02) and rust infestation rates (β = - 0.06, SE = 0.02) in AM communities. They were further significantly negatively related to rust infestation rates (β = - 0.07, SE = 0.02) in communities with both mycorrhizal types. Phenolic acid concentrations were significantly negatively related to total herbivory and herbivore mining rates (β = - 0.06, SE = 0.02 and β = - 0.02, SE = 0.01, respectively), whereas they were significantly positively related with rust infestation rates (β = 0.02, SE = 0.01) in AM tree communities.

In general, differences between proportions of variation explained by both metabolite group and the random factors (tree species identity and richness) and that explained by metabolite group only were lower in EM compared to AM communities (Appendix A: Table A.3). Similarly, differences were lower in regressions with phenolic acid concentrations and higher in regressions with flavone glucoside concentrations.

### SEM analysis of the effects of tree species richness and mycorrhizal type on leaf damage

We developed our final structural equation model by fitting eight models in total and removing the least important non-significant path in a stepwise approach (final model: χ^2^ = 26.56, df = 17, *P* = 0.06; CFI = 0.94, RMSEA = 0.07; CAIC = 305.44; see Appendix A: Table A.4 for results on each path).

**Fig. 3:**
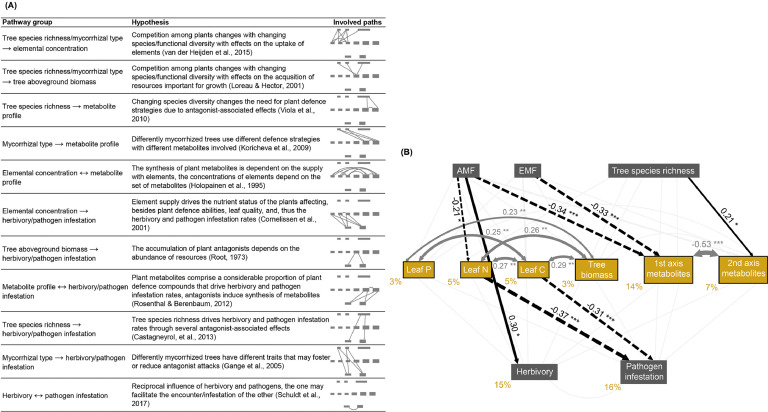
Predictions and results for the structural equation model (SEM) investigating the underlying mechanisms of the relationship between tree species richness and mycorrhizal type and herbivory and pathogen infestation rates. (A) Potential relationships between considered variables and the underlying hypotheses derived from previous studies. (B) The fitted model (SEM) including the two predictor variables, leaf element concentrations, tree aboveground biomass, the first two axes of the RDA based on metabolite profiles as affected by tree species richness and mycorrhizal type (with tree species identity partialled out) as well as herbivory and pathogen infestation rates (*N* = 107). Dark grey and black arrows represent significant relationships. Ochre-coloured values next to the endogenous variables indicate the variance explained. Black values and asterisks on the arrows indicate standardised path coefficients and significance, respectively: * *P* < 0.05, ** *P* < 0.01, *** *P* < 0.001. Coefficient values are reflected by the thickness of the arrow; solid arrows represent positive and dashed arrows negative coefficients. AMF: arbuscular mycorrhizal fungi, EMF: ectomycorrhizal fungi.

The model revealed that 15% and 16% of the variation in herbivory and pathogen infestation rates were explained by the predicting variables, respectively (Fig. 3B). The rates were not significantly correlated with each other. None of the rates were significantly related to metabolite profiles. Pathogen infestation rates were explained by C and N concentration, whereas herbivory rates were not explained by element concentrations. Pathogen infestation rates were negatively affected by leaf C (path coefficient: −0.31) as well as by leaf N (path coefficient: −0.37) concentration.

Tree species richness had only an impact on metabolite profiles (path coefficient second pRDA axis: 0.21). Both mycorrhizal types negatively affected the first pRDA axis of the metabolite profiles (path coefficient AM dominance: −0.34, EM dominance: −0.33). AM dominance (AM plots and plots with both mycorrhizal types) increased herbivory rates directly (path coefficient: 0.30). In contrast, pathogen infestation rates were only indirectly increased by AM dominance via a decrease in leaf N (path coefficient product: −0.21 x −0.37 = 0.08). EM dominance (EM plots and plots with both mycorrhizal types) did not affect damage rates.

Leaf elemental concentrations were not correlated with metabolite profiles, but they were positively correlated with tree biomass (path coefficient P: 0.23, N: 0.26, C: 0.29). Among the elements, leaf N and C (path coefficient: 0.27) as well as leaf P and C (path coefficient: 0.25) were positively correlated. Leaf P concentration neither affected herbivory nor pathogen infestation rates. The two pRDA axes of the metabolite profiles were negatively correlated (path coefficient: −0.53) but did not affect any of the rates.

## Discussion

Overall, our study showed that tree species identity was most important in driving leaf damage and chemical characteristics in tree saplings, especially in AM communities. Leaf elemental concentrations (C and N) and the presence of AM were significant predictors of leaf damage, whereas the concentrations of the assessed metabolites were not. Moreover, herbivory and pathogen infestation were explained by distinct relationships. These results indicate that aboveground multitrophic interactions depend on belowground associations of tree saplings with mycorrhiza and their related traits as well as their consequences for nutrient uptake.

### Effects of plant diversity on leaf damage

In contrast to the strong effects of tree species identity, the effects of tree species richness on total herbivory and pathogen infestation were not significant, but showed trends towards decreasing rates from monocultures to mixtures. Various underlying mechanisms have been proposed to explain this pattern, such as an increase of natural enemies of antagonists (Andow, 1991) and resource dilution (Castagneyrol et al., 2013; Hambäck, Inouye, Andersson & Underwood, 2014). Further, an enhanced resource-use complementarity and, thus, higher nutrient supply, that fosters synthesis of plant defence-related compounds, (Kostenko et al., 2017) may be responsible for the pattern. The lack of significant effects in our study might be explained by the short study duration. It may not have allowed for shifts in the abundance of natural enemies of antagonists, or shifts in resource uptake strategies among the tree species. Further, many parasitoids and predators are highly mobile, thereby making such effects less likely at the spatial scale of the experiment.

Leaf damage differed between tree communities of different mycorrhizal types. Specifically, the SEM showed that AM trees, i.e., in AM communities as well as in communities with both mycorrhizal types, increased total herbivory rates. This suggests that tree species associated with AM fungi share certain traits that facilitate attack by antagonists (Koele, Dickie, Oleksyn, Richardson & Reich, 2012; Koricheva, Gange & Jones, 2009). As indicated by the colonisation rates of AM and EM, in the AM trees, other traits than the degree of AM colonisation may have dominated the effects. Furthermore, we found that pathogen infestation rates were not directly affected by any of the mycorrhizal types and did not differ among tree species in EM communities. Thus pathogen-related traits seem to be more uniform across species than in communities that are associated with AM or both mycorrhizal types. In contrast to our expectation, tree communities with both mycorrhizal types experienced an intermediate rate of total herbivory and pathogen infestation that was in between that of AM and EM communities. We hypothesised that the effect of functionally diverse tree communities, e.g., communities that associate with different mycorrhizal types or occupy different ends of the leaf economics spectrum, adds to the complementarity in resource uptake of a species-diverse community (Barry et al., 2019; Eisenhauer, 2012; Wright et al., 2004). Such communities should therefore be better defended against antagonists (Eisenhauer et al., 2019; Meyer et al., 2016) by benefiting from the situation that interspecific competition is lower than intraspecific competition (Loreau & Hector 2001). However, our study indicates a pure additive effect, where the rate of leaf damage reflects the mixture of mycorrhizal types in the community. Moreover, with increasing mycorrhizal diversity, competitive relationships diverged between the two mycorrhizal types in the community, as did leaf damage. For example, leaf damage increased in AM trees and decreased in EM trees in communities with both mycorrhizal types (data not shown) as compared to communities with only one. Indeed, the SEM underpins the different roles and strategies of AM and EM trees in terms of resource supply.

### Drivers of leaf damage

C concentration was positively correlated with N and P concentration, which was unexpected as they are typically negatively or not correlated (Díaz et al., 2016; Bruelheide et al., 2018). Reasons for this may be related to the particularly high soil nutrient concentrations at the site (Ferlian et al., 2018b). Surprisingly, leaf elemental concentrations were not correlated with the profiles of the assessed leaf metabolites, as indicated by the SEM. Nutrients are commonly allocated to growth, defence, maintenance, reproduction, and storage within a plant (Chapin, Schulze & Mooney, 1990; Feng et al., 2009). However, investment in plant growth and defence are often suggested to be subject to a trade-off (McKey, 1974, 1979). Theories suggest that the resource quality of a habitat determines whether a plant invests predominantly into defence (lower antagonist infestation) or growth (lower competition; Cipollini et al., 2018; Eichenberg, Purschke, Ristok, Wessjohann & Bruelheide, 2015; Holopainen, Rikala, Kainulainen & Oksanen, 1995). Recent studies, however, indicate that plant growth and defence are not necessarily alternative strategies (Kempel, Schädler, Chrobock, Fischer & van Kleunen, 2011). In resource-rich habitats, such as our study site (Ferlian et al., 2018b), trees may have allocated nutrients preferably to other functions than chemical defence, such as growth. Therefore, plant nutrient status was mostly decoupled from defence-related metabolite concentrations. A further potential explanation could be that nutrients were allocated to secondary metabolites that were not measured in this study, such as volatile organic compounds.

Variation within the metabolite groups and profiles was mainly driven by tree species identity, whereas tree species richness and mycorrhizal type contributed similarly and only marginally after accounting for tree species. Metabolite profiles in monocultures generally only tended to differ from that of mixtures. Presence and concentrations of metabolites are highly species-specific representing defence strategies that species have evolved (Levin, 1976). In contrast, the high resource availability at the site may have decoupled metabolite concentrations from sources of nutrient input, such as mycorrhizal types. The different metabolite groups had mostly negative relationships with herbivory and pathogen infestation rates but effects were overall small suggesting a minor role of the secondary metabolites measured for leaf damage. The effects were even weaker for EM communities. Accordingly, in the SEM, metabolite profiles were not correlated with leaf damage. As mentioned above, due to the resource-rich habitat of the experimental site, herbivory and pathogen infestation rates may have been driven by other plant characteristics than by the assessed chemical defence compounds, especially in EM communities (Cornelissen, Aerts, Cerabolini, Werger & van der Heijden, 2001). Especially for leaf damage by pathogens, such characteristics may be related to nutrient status of the plant.

Surprisingly, the dominance of AM decreased leaf N status. This was unexpected as AM trees are typically characterised by a lower C-to-N ratio compared to EM trees (Lin, McCormack, Ma & Guo, 2017; Plett & Martin 2011; van der Heijden et al., 2015). Again, this may be attributed to the unique nutrient dynamics at the site and the young age of the trees presumably leading to weak or even detrimental interactions between the tree host and its symbiotic partner (Johnson, Graham & Smith, 1997). Leaf N concentrations, further, negatively affected pathogen infestation rates. This points to higher attraction of pathogens by AM trees due to additional relevant mechanisms driving pathogen infestation that involve N input but neither increased plant defence (at least not via the metabolites assessed in this study) nor leaf quality (Rabin & Pacovsky 1985). In contrast, the dominance of AM trees positively affected herbivory rates in a direct way suggesting that the effects on herbivory are mediated by other mechanisms than nutrient inputs all well. Such mechanisms may be related to enhanced water uptake, certain morphological traits, or antagonist-associated drivers. Path coefficients within the SEM suggest these positive direct effects to be much stronger than the positive effects through leaf N status (0.30 vs. 0.08).

Overall, the effects of AM dominance on the two antagonist groups were positive, whereas EM dominance did not determine them. This suggests that tree species associated with different mycorrhizal types may have evolved different functional characteristics that, to a different extent, influence insect herbivores and pathogens (Connell & Lowman 1989; Gehring, Cobb & Whitham, 1997).

### Mechanisms in herbivory vs. pathogen infestation

Our investigated variables and relationships explained a similar part of the variation in herbivory and pathogen infestation rates (15% and 16%, respectively). However, they were affected by different drivers (AM dominance and element concentrations, respectively), suggesting that underlying mechanisms of tree leaf damage by antagonists differ between insect herbivores and pathogens.

The SEM revealed that specific leaf elements determine pathogen infestation, whereas AM dominance and, thus, AM tree-associated characteristics determine herbivory (see above). Pathogen infestation was decreased by leaf N and C. The diverse relationships between leaf elemental concentrations and damage include a range of mechanisms, such as effects of chemical defences (that were not part of our set of assessed metabolites), and physical defences. Insect herbivores and pathogens substantially differ in characteristics like dietary needs, mobility, specialisation, enemy taxa, specificity to defence compounds, and susceptibility to physical barriers (Barrett & Heil 2012; Schuldt et al., 2017; Thaler, Agrawal & Halitschke, 2010). Thus, it is not surprising that they react differently to shifts in elemental concentrations.

Phenolic acid concentrations were negatively correlated with total herbivory and herbivore mining rates and positively correlated with rust infestation rates in AM communities, suggesting that here again opposing processes may have contributed to the relationships between phenolic acids and each total damage rate. However, most studies report negative relationships pointing to the important role of phenolic acids in constitutive and induced defence against herbivore and pathogens (Summers & Felton 1994).

Our study further showed no correlation between herbivory and pathogen infestation rates, which contrasts with previous studies (Schuldt et al., 2017; Stout, Thaler & Thomma, 2006). A multitude of studies has reported antagonistic as well as facilitative relationships between the two (Biere & Bennett 2013; Fernandez‐Conradi, Jactel, Robin, Tack & Castagneyrol, 2018; Schuldt et al., 2017). The strength of relationships between the two in trees also depends on the context, such as plant diversity and the dominating feeding guilds amongst the antagonist groups, with the latter being a potential explanation in our study (Schuldt et al., 2017; Stout et al., 2006). Moreover, the signalling pathways of anti-herbivore and anti-pathogen defences can interact antagonistically leading to opposing damage patterns in both groups (Pieterse, Schaller, Mauch-Mani & Conrath, 2006).

## Conclusions

We identified drivers of herbivory and pathogen infestation across tree monocultures and mixtures with different mycorrhizal associations. Including mycorrhizal type revealed a comparably minor role of tree species richness and functional richness in driving antagonist leaf damage in saplings, with tree species identity and AM dominance explaining most of the variation in herbivory and pathogen infestation rates. However, it has to be taken into consideration that the effects of mycorrhizal type are an interplay of the identity of the symbiotic partner and specific associated plant characteristics, mostly plant economics traits, that may co-determine and even contradict or countermand each other. Our study gives a first mechanistic insight into how those functionally distinct associations affect damage by herbivores and pathogens. However, our study also showed that species identity is a determinant of leaf damage by antagonists in tree saplings that have co-evolved with the multitude of plant strategies. Those effects cannot be attributed to a single process based on common ecological theories, but instead depend on a complex interplay of mechanisms. They involve host- as well as antagonist-associated processes that relate to the multifaceted characteristics of plant diversity. Furthermore, our study points to the importance of other mechanisms that are mediated via elemental concentrations, other than shifts in the measured defence-related metabolites. We speculate that shifts in physical defence and traits related to AM *per se* play a more crucial role in this regard than expected before. Furthermore, volatile organic compounds, that were not assessed in this study, may be more important in this context than the set of secondary metabolites measured. However, our study also showed relationships between the measured variables to be opposed to the ones commonly found. This may trigger a multitude of potential follow-up studies addressing such relationships in the light of different contexts. Our study further reveals how distinct the drivers of herbivory vs. pathogen infestation are in tree saplings with different mycorrhizal associations.

## Declaration of Competing Interest

The authors declare that they have no known competing financial interests or personal relationships that could have appeared to influence the work reported in this paper.

The authors declare the following financial interests/personal relationships which may be considered as potential competing interests

## Appendix Supplementary materials

Image, application 1

## References

[bib0001] Al-Alouni U., Brandl R., Auge H., Schädler M. (2014). Does insect herbivory on oak depend on the diversity of tree stands. Basic and Applied Ecology.

[bib0002] Altermann M., Rinklebe J., Merbach I., Körschens M., Langer U., Hofmann B. (2005). Chernozem - soil of the year 2005. Journal of Plant Nutrition and Soil Science.

[bib0003] Andow D.A. (1991). Vegetational diversity and arthropod population response. Annual Review of Entomology.

[bib0004] Barbosa P., Hines J., Kaplan I., Martinson H., Szczepaniec A., Szendrei Z. (2009). Associational resistance and associational susceptibility: having right or wrong neighbors. Annual Review of Ecology, Evolution, and Systematics.

[bib0005] Barrett L.G., Heil M. (2012). Unifying concepts and mechanisms in the specificity of plant–enemy interactions. Trends in Plant Science.

[bib0006] Barry K.E., Mommer L., van Ruijven J., Wirth C., Wright A.J., Bai Y. (2019). The future of complementarity: disentangling causes from consequences. Trends in Ecology and Evolution.

[bib0007] Barton K. (2013). MuMln: multi-model inference. R Package Version 1.9.5.

[bib0008] Bates D., Mächler M., Bolker B., Walker S. (2015). Fitting linear mixed-effects models using lme4. Journal of Statistical Software.

[bib0009] Biere A., Bennett A.E. (2013). Three-way interactions between plants, microbes and insects. Functional Ecology.

[bib0010] Bonfante P., Genre A. (2010). Mechanisms underlying beneficial plant–fungus interactions in mycorrhizal symbiosis. Nature Communications.

[bib0011] Bruelheide H., Dengler J., Purschke O., Lenoir J., Jiménez-Alfaro B., Hennekens S.M. (2018). Global trait–environment relationships of plant communities. Nature Ecology & Evolution.

[bib0012] Brundrett M.C. (2009). Mycorrhizal associations and other means of nutrition of vascular plants: Understanding the global diversity of host plants by resolving conflicting information and developing reliable means of diagnosis. Plant and Soil.

[bib0013] Cardinale B.J., Duffy J.E., Gonzalez A., Hooper D.U., Perrings C., Venail P. (2012). Biodiversity loss and its impact on humanity. Nature.

[bib0014] Castagneyrol B., Giffard B., Péré C., Jactel H. (2013). Plant apparency, an overlooked driver of associational resistance to insect herbivory. Journal of Ecology.

[bib0015] Cebrian J., Lartigue J. (2004). Patterns of herbivory and decomposition in aquatic and terrestrial ecosystems. Ecological Monographs.

[bib0016] Chapin F.S., Schulze E., Mooney H.A. (1990). The ecology and economics of storage in plants. Annual Review of Ecology and Systematics.

[bib0017] Cipollini D., Walters D., Voelckel C. (2018). Costs of resistance in plants: From theory to evidence. Annual Plant Reviews Online.

[bib0018] Connell J.H., Lowman M.D. (1989). Low-diversity tropical rain forests: some possible mechanisms for their existence. The American Naturalist.

[bib0019] Core Team R. (2014).

[bib0020] Cornelissen J., Aerts R., Cerabolini B., Werger M., van der Heijden M. (2001). Carbon cycling traits of plant species are linked with mycorrhizal strategy. Oecologia.

[bib0021] Díaz S., Kattge J., Cornelissen J.H., Wright I.J., Lavorel S., Dray S. (2016). The global spectrum of plant form and function. Nature.

[bib0022] Eichenberg D., Purschke O., Ristok C., Wessjohann L., Bruelheide H. (2015). Trade‐offs between physical and chemical carbon‐based leaf defence: Of intraspecific variation and trait evolution. Journal of Ecology.

[bib0023] Eisenhauer N. (2012). Aboveground–belowground interactions as a source of complementarity effects in biodiversity experiments. Plant and Soil.

[bib0024] Eisenhauer N., Barnes A.D., Cesarz S., Craven D., Ferlian O., Gottschall F. (2016). Biodiversity–ecosystem function experiments reveal the mechanisms underlying the consequences of biodiversity change in real world ecosystems. Journal of Vegetation Science.

[bib0025] Eisenhauer N., Bonkowski M., Brose U., Buscot F., Durka W., Ebeling A. (2019). Biotic interactions, community assembly, and eco-evolutionary dynamics as drivers of long-term biodiversity–ecosystem functioning relationships. Research Ideas and Outcomes: The Open Science Journal.

[bib0026] Feng Y.-.L., Lei Y.-.B., Wang R.-.F., Callaway R.M., Valiente-Banuet A., Inderjit (2009). Evolutionary tradeoffs for nitrogen allocation to photosynthesis versus cell walls in an invasive plant. Proceedings of the National Academy of Sciences.

[bib0027] Ferlian O., Biere A., Bonfante P., Buscot F., Eisenhauer N., Fernandez I. (2018). Growing Research Networks on Mycorrhizae for Mutual Benefits. Trends in Plant Science.

[bib0028] Ferlian O., Cesarz S., Craven D., Hines J., Barry K.E., Bruelheide H. (2018). Mycorrhiza in tree diversity–ecosystem function relationships: Conceptual framework and experimental implementation. Ecosphere (Washington, D.C).

[bib0029] Fernández I., Merlos M., López-Ráez J.A., Martínez-Medina A., Ferrol N., Azcón C. (2014). Defense Related Phytohormones Regulation in Arbuscular Mycorrhizal Symbioses Depends on the Partner Genotypes. Journal of Chemical Ecology.

[bib0030] Fernandez‐Conradi P., Jactel H., Robin C., Tack A.J., Castagneyrol B. (2018). Fungi reduce preference and performance of insect herbivores on challenged plants. Ecology.

[bib0031] Franceschi V.R., Krokene P., Christiansen E., Krekling T. (2005). Anatomical and chemical defenses of conifer bark against bark beetles and other pests. New Phytologist.

[bib0032] Gange A.C., West H.M. (1994). Interactions between arbuscular mycorrhizal fungi and foliar-feeding insects in *Plantago lanceolata* L. New Phytologist.

[bib0033] Gehring C.A., Cobb N.S., Whitham T.G. (1997). Three‐way interactions among ectomycorrhizal mutualists, scale insects, and resistant and susceptible pinyon pines. The American Naturalist.

[bib0034] Grace J.B. (2006).

[bib0035] Graniti A. (1998). Cypress canker: A pandemic in progress. Annual Review of Phytopathology.

[bib0036] Grossman J.J., Vanhellemont M., Barsoum N., Bauhus J., Bruelheide H., Castagneyrol B. (2018). Synthesis and future research directions linking tree diversity to growth, survival, and damage in a global network of tree diversity experiments. Environmental and Experimental Botany, 152.

[bib0037] Hambäck P.A., Inouye B.D., Andersson P., Underwood N. (2014). Effects of plant neighborhoods on plant–herbivore interactions: Resource dilution and associational effects. Ecology.

[bib0038] Hantsch L., Bien S., Radatz S., Braun U., Auge H., Bruelheide H. (2014). Tree diversity and the role of non-host neighbour tree species in reducing fungal pathogen infestation. Journal of Ecology.

[bib0039] Hines J., van der Putten W.H., De Deyn G.B., Wagg C., Voigt W., Mulder C., Woodward G., Bohan D.A. (2015).

[bib0040] Holopainen J.K., Rikala R., Kainulainen P., Oksanen J. (1995). Resource partitioning to growth, storage and defence in nitrogen-fertilized Scots pine and susceptibility of the seedlings to the tarnished plant bug Lygus rugulipennis. New Phytologist.

[bib0041] Jactel H., Brockerhoff E.G. (2007). Tree diversity reduces herbivory by forest insects. Ecology Letters.

[bib0042] Johnson N.C., Graham J.H., Smith F.A. (1997). Functioning of mycorrhizal associations along the mutualism–parasitism continuum. The New Phytologist.

[bib0043] Jouveau S., Toïgo M., Giffard B., Castagneyrol B., Van Halder I., Vétillard F. (2020). Carabid activity‐density increases with forest vegetation diversity at different spatial scales. Insect Conservation and Diversity.

[bib0044] Kaling M., Schmidt A., Moritz F., Rosenkranz M., Witting M., Kasper K. (2018). Mycorrhiza-triggered transcriptomic and metabolomic networks impinge on herbivore fitness. Plant Physiology.

[bib0045] Kempel A., Schädler M., Chrobock T., Fischer M., van Kleunen M. (2011). Tradeoffs associated with constitutive and induced plant resistance against herbivory. Proceedings of the National Academy of Sciences.

[bib0046] Kempel A., Schmidt A.K., Brandl R., Schädler M. (2010). Support from the underground: Induced plant resistance depends on arbuscular mycorrhizal fungi. Functional Ecology.

[bib0047] Klironomos J.N., McCune J., Hart M., Neville J. (2000). The influence of arbuscular mycorrhizae on the relationship between plant diversity and productivity. Ecology Letters.

[bib0048] Koele N., Dickie I.A., Oleksyn J., Richardson S.J., Reich P.B. (2012). No globally consistent effect of ectomycorrhizal status on foliar traits. New Phytologist.

[bib0049] Koricheva J., Gange A.C., Jones T. (2009). Effects of mycorrhizal fungi on insect herbivores: A meta-analysis. Ecology.

[bib0050] Kostenko O., Mulder P.P.J., Courbois M., Bezemer T.M. (2017). Effects of plant diversity on the concentration of secondary plant metabolites and the density of arthropods on focal plants in the field. Journal of Ecology.

[bib0051] Levin D.A. (1976). The chemical defenses of plants to pathogens and herbivores. Annual Review of Ecology and Systematics.

[bib0052] Lin G., McCormack M.L., Ma C., Guo D. (2017). Similar below-ground carbon cycling dynamics but contrasting modes of nitrogen cycling between arbuscular mycorrhizal and ectomycorrhizal forests. New Phytologist.

[bib0053] Loreau M., Hector A. (2001). Partitioning selection and complementarity in biodiversity experiments. Nature.

[bib0054] Martinez-Medina A., Flors V., Heil M., Mauch-Mani B., Pieterse C.M.J., Pozo M.J. (2016). Recognizing plant defense priming. Trends in Plant Science.

[bib0055] McKey D. (1974). Adaptive patterns in alkaloid physiology. The American Naturalist.

[bib0056] McKey D. (1979). Herbivores-their interaction with secondary plant metabolites.

[bib0057] Meyer S.T., Ebeling A., Eisenhauer N., Hertzog L., Hillebrand H., Milcu A. (2016). Effects of biodiversity strengthen over time as ecosystem functioning declines at low and increases at high biodiversity. Ecosphere (Washington, D.C).

[bib0058] Mraja A., Unsicker S.B., Reichelt M., Gershenzon J., Roscher C. (2011). Plant community diversity influences allocation to direct chemical defence in Plantago lanceolata. PloS One.

[bib0059] Nguyen D., Castagneyrol B., Bruelheide H., Bussotti F., Guyot V., Jactel H. (2016). Fungal disease incidence along tree diversity gradients depends on latitude in European forests. Ecology and Evolution.

[bib0060] Oelmann Y., Wilcke W., Temperton V.M., Buchmann N., Roscher C., Schumacher J. (2007). Soil and plant nitrogen pools as related to plant diversity in an experimental grassland. Soil Science Society of America Journal.

[bib0061] Oksanen J., Blanchet F.G., Kindt R., Legendre P., Minchin P.R., O'hara R.B. (2013). Package ‘vegan’. Community Ecology Package.

[bib0062] Pieterse C.M.J., Schaller A., Mauch-Mani B., Conrath U., Tuzun S., Bent E. (2006). Multigenic and induced systemic resistance in plants.

[bib0063] Plett J.M., Martin F. (2011). Blurred boundaries: Lifestyle lessons from ectomycorrhizal fungal genomes. Trends in Genetics.

[bib0064] Rabin L.B., Pacovsky R.S. (1985). Reduced larva growth of two lepidoptera (Noctuidae) on excised leaves of soybean infected with a mycorrhizal fungus1. Journal of Economic Entomology.

[bib0065] Root R.B. (1973). Organization of a plant-arthropod association in simple and diverse habitats: the fauna of collards (Brassica Oleracea). Ecological Monographs.

[bib0066] Schuldt A., Baruffol M., Bohnke M., Bruelheide H., Hardtle W., Lang A.C. (2010). Tree diversity promotes insect herbivory in subtropical forests of south-east China. Journal of Ecology.

[bib0067] Schuldt A., Hönig L., Li Y., Fichtner A., Härdtle W., von Oheimb G. (2017). Herbivore and pathogen effects on tree growth are additive, but mediated by tree diversity and plant traits. Ecology and Evolution.

[bib0068] Smith S.E., Read D.J. (2010).

[bib0069] Soudzilovskaia N.A., van Bodegom P.M., Terrer C., Zelfde M.v.t., McCallum I., Luke McCormack M. (2019). Global mycorrhizal plant distribution linked to terrestrial carbon stocks. Nature Communications.

[bib0070] Stamp N. (2003). Out of the quagmire of plant defense hypotheses. The Quarterly Review of Biology.

[bib0071] Sterner R.W., Elser J.J., Fee E.J., Guildford S.J., Chrzanowski T.H. (1997). The light: Nutrient ratio in lakes: the balance of energy and materials affects ecosystem structure and process. The American Naturalist.

[bib0072] Stout M.J., Thaler J.S., Thomma B.P.H.J. (2006). Plant-mediated interactions between pathogenic microorganisms and herbivorous arthropods. Annual Review of Entomology.

[bib0073] Summers C.B., Felton G.W. (1994). Prooxidant effects of phenolic acids on the generalist herbivore *Helicoverpa zea* (Lepidoptera: Noctuidae): Potential mode of action for phenolic compounds in plant anti-herbivore chemistry. Insect Biochemistry and Molecular Biology.

[bib0074] Thaler J.S., Agrawal A.A., Halitschke R. (2010). Salicylate-mediated interactions between pathogens and herbivores. Ecology.

[bib0075] Unsicker S.B., Oswald A., Köhler G., Weisser W.W. (2008). Complementarity effects through dietary mixing enhance the performance of a generalist insect herbivore. Oecologia.

[bib0076] van der Heijden M.G., Martin F.M., Selosse M.A., Sanders I.R. (2015). Mycorrhizal ecology and evolution: The past, the present, and the future. New Phytologist.

[bib0077] Vehviläinen H., Koricheva J., Ruohomäki K. (2007). Tree species diversity influences herbivore abundance and damage: Meta-analysis of long-term forest experiments. Oecologia.

[bib0078] Wagg C., Barendregt C., Jansa J., van der Heijden M.G.A. (2015). Complementarity in both plant and mycorrhizal fungal communities are not necessarily increased by diversity in the other. Journal of Ecology.

[bib0079] Wang B., Qiu Y.L. (2006). Phylogenetic distribution and evolution of mycorrhizas in land plants. Mycorrhiza.

[bib0080] Weisser W.W., Siemann E., Weisser W.W., Siemann E. (2008). Insects and ecosystem function.

[bib0081] White J.A., Whitham T.G. (2000). Associational susceptibility of cottonwood to a box elder herbivore. Ecology.

[bib0082] Wickham H. (2016).

[bib0083] Wright I.J., Reich P.B., Westoby M., Ackerly D.D., Baruch Z., Bongers (2004). The worldwide leaf economics spectrum. Nature.

