# Supplementary material for: Nutrient status not secondary metabolites drives herbivory and pathogen infestation across differently mycorrhized tree monocultures and mixtures
Source: Basic Appl Ecol. Author manuscript; Available in PMC 2021 Sep 6. (PMC7611625; doi:10.1016/j.baae.2020.09.009)
Supplement: Supplementary file 1 [file EMS117820-supplement-Appendix_A.docx]

**Appendix A for Ferlian et al. entitled “Nutrient status not secondary metabolites drives herbivory and pathogen infestation across differently mycorrhized tree monocultures and mixtures”**

**Figure A.1.** Mycorrhizal colonisation frequencies of (A) arbuscular mycorrhiza (AM), (B) ectomycorrhiza (EM), (C) the ratio of AM and EM in the five tree species typically associated with AM fungi (green boxes) and that typically associated with EM fungi (blue boxes). Letters indicate contrasts between groups according to Tukey’s honestly significant difference test. *Acer pseudoplatanus:* Ac, *Aesculus hippocastanum:* Ae, *Fraxinus excelsior*: Fr, *Prunus avium*: Pr, *Sorbus aucuparia*: So, *Betula pendula*: Be, *Carpinus betulus*: Ca, *Fagus sylvatica*: Fa, *Quercus petraea*: Qu, *Tilia platyphyllos*: Ti.

**Methods A.1.** Assessments of mycorrhizal colonisation rates

In September 2016, trees were harvested by cutting them at the soil surface to separate roots from shoots. The root part was dug out in a cubical form (1 x 1 m) around the main stem. Fine roots were randomly sampled from the individual trees from different main roots per tree. While all four trees in a plot were sampled in mixtures, only two trees were sampled per plot in monocultures.

The samples were cleaned with tap water and divided up into root fragments for analysis of ectomycorrhizal (EM) and arbuscular mycorrhizal (EM) colonisation rates. The EM samples were placed into sample tubes and filled with tap water, while the AM samples were stored in sample tubes containing an ethanol solution (50% ethanol). The samples were stored at 4 °C until further processing. 

For assessing the mycorrhization with AM fungi, the samples were stained with Trypan blue following the protocol set out by (Chabaud et al. 2006). First, the tubes were filled with a potassium hydroxide solution (10% KOH), heated to 90 °C, and kept for two hours. Then, the roots were drained and washed. The roots were then bleached for 1.5-2 hours using a hydrogen peroxide solution (10% H_2_O_2_) containing a few drops of ammonia (25% NH_3_). Subsequently, the solution was drained, roots were washed, tubes were refilled, a few drops of highly concentrated lactic acid (>85% C_3_H_6_O_3_) were added, and kept for one hour. The lactic acid solution was then drained and the tubes were filled with a lactoglycerol solution (300 ml 50% glycerol solution, 300 ml lactic acid (>85%) and 400 ml distilled water) containing 25 ml of 2% Trypan blue solution and heated to 100 °C for 3 minutes. Finally, the dye was drained, the tubes were filled with pure lactoglycerol and stored in a fridge.

The assessment of EM was conducted using a stereo microscope (Stemi DV 4; Zeiss, Jena, Germany). Five 3 cm long root pieces were measured, trimmed and placed in a petri dish. The root tips of each fragment were counted distinguishing between roots showing no or only inactive ectomycorrhizal colonisation and those showing active ectomycorrhizal structures. Frequency of active ectomycorrhizal root tips was expressed in percent of active EM.

The assessment of AM was conducted using thirty 1-cm-root fragments per sample. Using a light microscope (Axiostar Plus; Zeiss, Jena, Germany), we assessed AM colonisation rates as frequencies of mycorrhiza following Trouvelot et al. (1986). We used AM and EM frequencies gained from the two approaches to calculate the AM-to-EM frequency ratio. This gave us a quantitative measure of colonisation rates combining both parameters.

References

Chabaud, M., Harrison, M., de Carvalho-Niebel, F., Bécard, G., & Barker, D. G. (2006). Inoculation and growth of Mycorrhizal fungi. The *Medicago truncatula* handbook.

Trouvelot, A., Kough, J.L., & Gianinazzi-Pearson, V. (1986). Mesure du taux de mycorhization VA d’un systeme radiculaire. Recherche de methodes d’estimation ayant une signification fonctionnelle. Physiological and genetical aspects of mycorrhizae - Aspects physiologiques et genetiques des mycorhizes. Proceedings of the 1st European Symposium on Mycorrhizae, Dijon, 1-5 July 1985 (pp. 217-221).

**Methods A.2.** Analyses of leaf chemical compounds

Amino acid analysis 

Amino acids were quantified with an LC-MS/MS on a C18-column (XDB-C18, 50 x 4.6 mm x 1.8 µm; Agilent, Santa Clara, CA, USA) after diluting the methanol extracts 1:10 with water containing 10 µg ml-1 of a mixture of 15N/13C labelled amino acids (Isotec, Miamisburg, OH, USA). For details on the chromatography and mass spectrometry, see Crocoll, Halkier, & Burow (2016). All amino acids were quantified relative to the peak area of the corresponding labelled compound, except for tryptophan (using phenylalanine and applying a response factor of 0.42) and asparagine (using aspartate and a response factor of 1.0).

Sugar analysis

Soluble sugars were analysed from the methanol extracts (at 1:10 dilution in water), by LC-MS/MS on a hydrophilic interaction liquid chromatography (HILIC) column (apHera-NH2 Polymer; Supelco, Bellefonte, PA, USA) as described in Madsen et al. (2015). All sugars were quantiﬁed using an external standard curve with authentic standards of glucose, fructose, sucrose, stachyose (all from Sigma-Aldrich) and rafﬁnose (Fluka, Seelze, Germany).

Analysis of phenolic compounds 

Chromatographic separation of phenolic compounds in leaf extracts of the different tree species was achieved using an Agilent 1260 infinity II LC system (Agilent, Santa Clara, USA) equipped with a Zorbax Eclipse XDB-C18 column (50 × 4.6 mm, 1.8 μm, Agilent), using aqueous formic acid (0.05% (v/v)) and acetonitrile as mobile phases A and B, respectively. The mobile phase flow rate was 1.1 ml/min. The elution profile was: 0-0.5 min, 5% B; 0.5-6.0 min, 5-37.4% B; 6.02-7.5 min, 80-100% B; 7.5-9.5 min, 100% B; 9.52-12 min, 5% B. The column temperature was maintained at 20 °C. The LC system was coupled to a QTRAP 6500 tandem mass spectrometer (Sciex, Darmstadt, Germany) equipped with a turbospray ion source, operated in negative ionization mode. Ion spray voltage was maintained at -4500 eV and the turbo gas temperature was set at 650 °C. Nebulising gas was set at 60 psi, curtain gas at 40 psi, heating gas at 60 psi, and collision gas at medium level. Multiple reaction monitoring (MRM) was used to monitor analyte parent ion - product ion formation (Table A.2.).. Analyst 1.6.3 software (Applied Biosystems, Foster City, USA) was used for data acquisition and processing.

References

Crocoll, C., Mirza, N., Reichelt, M., Gershenzon, J., & Halkier, B. A. (2016). Optimization of engineered production of the glucoraphanin precursor dihomomethionine in *Nicotiana benthamiana*. Frontiers in Bioengineering and Biotechnology, 4, 14. doi:10.3389/fbioe.2016.00014

Madsen, S. R., Kunert, G., Reichelt, M., Gershenzon, J., & Halkier, B. A. (2015). Feeding on leaves of the glucosinolate transporter mutant gtr1gtr2 reduces fitness of *Myzus persicae*. Journal of Chemical Ecology, 41(11), 975-984. doi:10.1007/s10886-015-0641-3

**Table A.1.** Details on analysis of phenolics by LC-MS/MS [HPLC 1260 (Agilent Technologies)-QTRAP6500 (SCIEX)] in negative ionisation mode.

| Compound | Q1 | Q3 | Retention time (min) | Declustering potential | Entrance potential | Collision energy | Collision cell exit potential |
| --- | --- | --- | --- | --- | --- | --- | --- |
| 2,3 Dihydroxybenzoic acid | 153.01 | 108 | 4 | -20 | -8 | -28 | -15 |
| 2,3 Dihydroxybenzoic acid-glucose* | 315.01 | 108 | 2.4 | -20 | -10 | -28 | -3 |
| 2,3 Dihydroxybenzoic acid-glucoside* | 315.01 | 109 | 2.4 | -20 | -10 | -28 | -3 |
| 2,5 Dihydroxy-benzoic acid | 153 | 108 | 3.6 | -20 | -8 | -28 | -15 |
| 2,5 Dihydroxybenzoic acid-glucoside* | 315 | 108 | 1.8 | -20 | -10 | -28 | -3 |
| 2,5 Dihydroxybenzoic acid-glucoside* | 315 | 109 | 1.8 | -20 | -10 | -28 | -3 |
| 2,5 Dihydroxybenzoic acid-glucoside* | 315 | 153 | 1.8 | -20 | -10 | -18 | -3 |
| 2-Hydroxycinnamic acid | 163.1 | 119 | 5.7 | -20 | -8 | -20 | -5 |
| 3-Caffeoylquinic acid* | 353.02 | 179.1 | 3.2 | -20 | -4 | -22 | -4 |
| 3-Hydroxybenzoic acid | 137.01 | 93.01 | 4.1 | -20 | -8 | -16 | -2 |
| 3-Hydroxybenzylalcohol | 123.01 | 93.01 | 3.01 | -20 | -10 | -16 | -5 |
| 4-Caffeoylquinic acid* | 353.1 | 173 | 3.9 | -20 | -4 | -22 | -4 |
| 4-Hydroxybenzoic acid | 137 | 93 | 3.6 | -20 | -2.5 | -20 | 0 |
| 4-Hydroxybenzylalcohol | 123.02 | 105 | 2.37 | -20 | -10 | -14 | -11 |
| 4-Hydroxybenzylalcohol | 123.02 | 77 | 2.37 | -20 | -10 | -22 | -9 |
| 5-Caffeoylquinic acid* | 353 | 190.9 | 3.9 | -20 | -4 | -22 | -4 |
| Apigenin-glucoside | 431 | 268 | 5.57 | -20 | -8 | -44 | -5 |
| Benzoic acid | 121 | 121 | 5.6 | -20 | -10 | -5 | -10 |
| Caffeic acid | 179 | 134.9 | 4 | -20 | -8 | -22 | -5 |
| Catechin | 288.8 | 109.1 | 3.7 | -20 | -11 | -32 | -2 |
| Coumaric acid | 163 | 118.9 | 4.7 | -20 | -8 | -20 | -5 |
| Dihydroxybenzoic acid (Protocatechuic acid) | 153 | 108.001 | 2.69 | -20 | -8 | -28 | -5 |
| Ferulic acid | 193.1 | 133.9 | 5 | -20 | -8 | -22 | -5 |
| Gallic acid | 169 | 125 | 1.3 | -20 | -10 | -18 | -19 |
| Gallocatechin | 304.9 | 125 | 3 | -20 | -8 | -31 | -5 |
| Isoscopoletin | 191 | 176 | 4.8 | -20 | -7.5 | -18 | -4 |
| Luteolin-di-glucoside | 609 | 447 | 4.65 | -20 | -8 | -34 | -5 |
| Luteolin-glucoside | 447 | 285 | 5.11 | -20 | -8 | -40 | -5 |
| Proanthocyanidin B1 | 576.9 | 289.1 | 3.56 | -20 | -9 | -38 | -4 |
| Proanthocyanidin B1-3 | 576.91 | 289.1 | 4.26 | -20 | -9 | -38 | -4 |
| Salicylalcohol | 123 | 93 | 3.5 | -20 | -10 | -22 | -2 |
| Salicylalcohol (unknown)2* | 123.01 | 93 | 2.85 | -20 | -10 | -22 | -2 |
| Salicylic acid-glucoside | 299.128 | 136.9 | 3.3 | -20 | -10 | -18 | -21 |
| Scopoletin | 191.01 | 176 | 5.12 | -20 | -8 | -18 | -5 |
| Syringic acid | 197 | 121.085 | 4.14 | -20 | -8 | -24 | -5 |
| Umbelliferone | 161 | 133 | 5 | -20 | -9 | -26 | -2 |
| Vanillic acid | 167 | 123 | 4 | -20 | -8 | -18 | -5 |
| Vitexin | 431 | 311 | 4.94 | -20 | -8 | -32 | -5 |

**Table A.2.** List of leaf metabolites analysed within the study.

| **Metabolite group** | **Metabolite** |
| --- | --- |
| Amino acid | Alanine, Serine, Proline, Valine, Threonine, Isoleucine, Leucine, Aspartic acid, Glutamine, Methionine, Histidine, Phenylalanine, Arginine, Tyrosine, Tryptophan, Asparagine, Glutamine, Lysine |
|  |  |
| Sugar | Glucose, Fructose, Sucrose, Trisaccharide, Tetrasaccharide, Pentasaccharide |
| Benzyl alcohol derivative | 3-Hydroxybenzylalcohol, 4-Hydroxybenzylalcohol, 4-Hydroxybenzylalcohol, Salicylalcohol, Salicylalcohol (unknown)^2*^ |
| Coumarin | Isoscopoletin, Scopoletin, Umbelliferone |
| Flavan-3-ol | Catechin, Gallocatechin, Proanthocyanidin B1, Proanthocyanidin B1-3 |
| Flavone glucoside | Apigenin-glucoside, Luteolin-glucoside, Luteolin-di-glucoside, Vitexin |
| Phenolic acid | 3-Caffeoylquinic acid, 4-Caffeoylquinic acid, 5-Caffeoylquinic acid, 2,3 Dihydroxybenzoic acid, 2,5 Dihydroxy-benzoicacid, 2-Hydroxycinnamic acid, 3-Hydroxybenzoic acid, 4-Hydroxybenzoic acid, Benzoic acid, Caffeic acid, Coumaric acid, Dihydroxybenzoic acid (Protocatechuic acid), Ferulic acid, Gallic acid, 2,3 Dihydroxybenzoic acid-glucoside*, 2,3 Dihydroxybenzoic acid-glucose*, 2,5 Dihydroxybenzoic acid-glucoside*, 2,5 Dihydroxybenzoic acid-glucoside*, 2,5 Dihydroxybenzoic acid-glucoside*, Salicylic acid-glucoside, Syringic acid, Vanillic acid |
| *compounds not verified with NMR, only putative |  |

**Figure A.2.** Ordination biplot of the partial redundancy analysis (pRDA) illustrating the relationship between metabolite profiles and tree species richness and mycorrhizal type (as composite variable) after partialling out tree species identity. Mono: monoculture, Mix: mixture.

**Table A.3.** Summary of linear mixed eﬀects analyses of regressions between secondary metabolite concentrations and total and feeding type-specific herbivory and pathogen infestation rates (fixed factor). The variables tree species identity and tree species richness were used as random factors. Analyses were conducted separately for AM, EM, and communities with both mycorrhizal types. Estimates (β) represent the change. Marginal (only fixed effects, R^2^_m_) and conditional (fixed and random effects, R^2^_c_) R^2^-values are given. Signiﬁcant eﬀects (P < 0.05) are given in bold (df = 1).

|  |  | AM |  |  |  |  |  | EM |  |  |  |  |  | Both |  |  |  |  |
| --- | --- | --- | --- | --- | --- | --- | --- | --- | --- | --- | --- | --- | --- | --- | --- | --- | --- | --- |
|  |  | β | SE | χ^2^ | R^2^_m_ | R^2^_c_ |  | β | SE | χ^2^ | R^2^_m_ | R^2^_c_ |  | β | SE | χ^2^ | R^2^_m_ | R^2^_c_ |
| Total herbivory | Benzyl | -0.08 | 0.08 | 1.09 | 0.03 | 0.81 |  | 0.02 | 0.10 | 0.14 | 0.00 | 0.31 |  | -0.05 | 0.06 | 0.70 | 0.01 | 0.63 |
| Hole-feeder | alcohol | 0.00 | 0.02 | < 0.01 | < 0.01 | 0.83 |  | 0.05 | 0.03 | 2.56 | 0.07 | 0.30 |  | -0.01 | 0.02 | 0.22 | < 0.01 | 0.63 |
| Miner | derivatives | **-0.05** | **0.02** | **5.90** | **0.18** | **0.82** |  | 0.03 | 0.04 | 0.62 | 0.02 | 0.29 |  | -0.02 | 0.02 | 0.75 | 0.01 | 0.61 |
| Total pathogens |  | 0.03 | 0.05 | 0.40 | < 0.01 | 0.83 |  | -0.02 | 0.10 | 0.01 | 0.00 | 0.32 |  | 0.00 | 0.06 | < 0.01 | < 0.01 | 0.63 |
| Rust |  | **-0.06** | **0.02** | **3.90** | **0.07** | **0.91** |  | 0.05 | 0.10 | 0.29 | 0.01 | 0.29 |  | **-0.07** | **0.02** | **6.35** | **0.09** | **0.80** |
| Mildew |  | 0.06 | 0.05 | 1.19 | 0.01 | 0.84 |  | 0.07 | 0.09 | 0.89 | 0.02 | 0.29 |  | 0.01 | 0.07 | 0.02 | < 0.01 | 0.63 |
|  |  |  |  |  |  |  |  |  |  |  |  |  |  |  |  |  |  |  |
| Total herbivory | Coumarins | 0.07 | 0.12 | 0.25 | 0.01 | 0.72 |  | 0.02 | 0.23 | 0.05 | < 0.01 | 0.41 |  | -0.03 | 0.15 | 0.02 | < 0.01 | 0.26 |
| Hole-feeder |  | 0.03 | 0.03 | 1.10 | 0.02 | 0.70 |  | < 0.01 | 0.07 | < 0.01 | < 0.01 | 0.40 |  | -0.05 | 0.05 | 1.15 | 0.02 | 0.28 |
| Miner |  | 0.01 | 0.04 | 0.08 | < 0.01 | 0.71 |  | 0.01 | 0.09 | 0.04 | < 0.01 | 0.40 |  | 0.01 | 0.05 | 0.01 | < 0.01 | 0.25 |
| Total pathogens |  | 0.06 | 0.08 | 0.55 | 0.01 | 0.70 |  | 0.12 | 0.23 | 0.37 | 0.01 | 0.38 |  | 0.02 | 0.17 | 0.02 | < 0.01 | 0.25 |
| Rust |  | 0.04 | 0.04 | 0.96 | 0.03 | 0.72 |  | 0.04 | 0.24 | 0.05 | < 0.01 | 0.40 |  | -0.13 | 0.06 | 3.75 | 0.09 | 0.36 |
| Mildew |  | -0.15 | 0.08 | 3.54 | 0.04 | 0.72 |  | -0.09 | 0.20 | 0.17 | < 0.01 | 0.42 |  | -0.01 | 0.19 | < 0.01 | < 0.01 | 0.24 |
|  |  |  |  |  |  |  |  |  |  |  |  |  |  |  |  |  |  |  |
| Total herbivory | Flavan- | -0.17 | 0.19 | 0.61 | 0.03 | 0.82 |  | -0.05 | 0.14 | 0.14 | < 0.01 | < 0.01 |  | -0.13 | 0.12 | 1.33 | 0.02 | 0.68 |
| Hole-feeder | 3-ols | -0.02 | 0.04 | 0.30 | < 0.01 | 0.78 |  | 0.03 | 0.05 | 0.35 | 0.01 | 0.01 |  | 0.02 | 0.04 | 0.42 | < 0.01 | 0.68 |
| Miner |  | -0.08 | 0.05 | 1.93 | 0.06 | 0.83 |  | 0.01 | 0.06 | 0.01 | < 0.01 | < 0.01 |  | -0.05 | 0.04 | 1.66 | 0.02 | 0.70 |
| Total pathogens |  | 0.07 | 0.13 | 0.31 | < 0.01 | 0.77 |  | -0.07 | 0.16 | 0.22 | 0.01 | 0.01 |  | 0.04 | 0.13 | 0.08 | < 0.01 | 0.68 |
| Rust |  | < 0.01 | 0.06 | < 0.01 | < 0.01 | 0.78 |  | -0.10 | 0.17 | 0.37 | 0.01 | 0.01 |  | -0.06 | 0.05 | 0.92 | < 0.01 | 0.72 |
| Mildew |  | 0.22 | 0.13 | 3.02 | 0.03 | 0.79 |  | 0.08 | 0.15 | 0.22 | 0.01 | 0.04 |  | -0.06 | 0.14 | 0.24 | < 0.01 | 0.68 |
|  |  |  |  |  |  |  |  |  |  |  |  |  |  |  |  |  |  |  |
| Total herbivory | Flavone | -0.10 | 0.09 | 0.63 | 0.03 | 0.87 |  | -0.06 | 0.09 | 0.43 | < 0.01 | 0.86 |  | 0.05 | 0.07 | 0.49 | < 0.01 | 0.84 |
| Hole-feeder | glucosides | -0.02 | 0.02 | 1.39 | 0.01 | 0.83 |  | 0.02 | 0.03 | 0.32 | < 0.01 | 0.86 |  | -0.01 | 0.02 | 0.29 | < 0.01 | 0.84 |
| Miner |  | -0.05 | 0.03 | 2.43 | 0.06 | 0.88 |  | -0.03 | 0.03 | 0.68 | < 0.01 | 0.85 |  | < 0.01 | 0.02 | < 0.01 | < 0.01 | 0.84 |
| Total pathogens |  | -0.01 | 0.06 | 0.01 | < 0.01 | 0.81 |  | -0.12 | 0.09 | 1.79 | 0.01 | 0.87 |  | 0.03 | 0.08 | 0.12 | < 0.01 | 0.84 |
| Rust |  | -0.02 | 0.03 | 0.59 | 0.01 | 0.82 |  | -0.10 | 0.09 | 1.12 | 0.01 | 0.87 |  | -0.01 | 0.03 | 0.17 | < 0.01 | 0.85 |
| Mildew |  | 0.08 | 0.06 | 1.66 | 0.01 | 0.81 |  | 0.01 | 0.08 | 0.01 | < 0.01 | 0.86 |  | -0.01 | 0.08 | 0.03 | < 0.01 | 0.84 |
|  |  |  |  |  |  |  |  |  |  |  |  |  |  |  |  |  |  |  |
| Total herbivory | Phenolic | **-0.06** | **0.02** | **6.41** | **0.18** | **0.26** |  | 0.04 | 0.10 | 0.26 | 0.01 | 0.41 |  | -0.02 | 0.07 | 0.09 | < 0.01 | < 0.01 |
| Hole-feeder | acids | 0.01 | 0.01 | 1.35 | 0.03 | 0.17 |  | 0.05 | 0.03 | 2.61 | 0.06 | 0.44 |  | -0.01 | 0.03 | 0.14 | < 0.01 | < 0.01 |
| Miner |  | **-0.02** | **0.01** | **5.41** | **0.16** | **0.29** |  | 0.01 | 0.04 | 0.13 | < 0.01 | 0.42 |  | -0.01 | 0.02 | 0.23 | < 0.01 | < 0.01 |
| Total pathogens |  | 0.02 | 0.03 | 0.78 | 0.02 | 0.18 |  | -0.01 | 0.10 | 0.01 | < 0.01 | 0.43 |  | -0.02 | 0.08 | 0.07 | < 0.01 | < 0.01 |
| Rust |  | **0.02** | **0.01** | **4.09** | **0.12** | **0.19** |  | < 0.01 | 0.10 | 0.01 | < 0.01 | 0.43 |  | -0.01 | 0.03 | 0.12 | < 0.01 | < 0.01 |
| Mildew |  | 0.01 | 0.04 | 0.21 | < 0.01 | 0.20 |  | 0.02 | 0.09 | 0.05 | < 0.01 | 0.42 |  | 0.02 | 0.10 | 0.04 | < 0.01 | < 0.01 |

**Table A.4.** Standardized regression weights (Estimate [Std]), unstandardized regression weights (Estimate), standard errors (SE), critical ratio (C.R.) and P-value of the paths in the fitted model. Significant relationships are given in bold; *** P < 0.001.

| Path |  |  | Estimate (Std) | Estimate | SE | C.R. | P |
| --- | --- | --- | --- | --- | --- | --- | --- |
| EM | -> | Leaf C | 0.09 | 0.22 | 0.22 | 0.98 | 0.33 |
| AM | -> | Leaf C | -0.17 | -0.39 | 0.22 | -1.79 | 0.07 |
| Tree species richness | -> | Tree biomass | 0.08 | 0.21 | 0.23 | 0.91 | 0.36 |
| EM | -> | Leaf P | -0.15 | -0.34 | 0.23 | -1.49 | 0.14 |
| AM | -> | Leaf P | 0.06 | 0.14 | 0.22 | 0.63 | 0.53 |
| **AM** | **->** | **Leaf N** | **-0.21** | **-0.47** | **0.20** | **-2.31** | **0.02** |
| Tree species richness | -> | Leaf N | 0.08 | 0.20 | 0.23 | 0.86 | 0.39 |
| **Leaf N** | **->** | **Pathogen infestation rate** | **-0.37** | **-0.37** | **0.09** | **-4.25** | ******* |
| **Leaf C** | **->** | **Pathogen infestation rate** | **-0.31** | **-0.31** | **0.09** | **-3.49** | ******* |
| Leaf C | -> | Herbivory rate | 0.14 | 0.14 | 0.10 | 1.39 | 0.17 |
| Leaf N | -> | Herbivory rate | 0.23 | 0.23 | 0.10 | 1.39 | 0.17 |
| Leaf P | -> | Pathogen infestation rate | 0.09 | 0.09 | 0.09 | 1.02 | 0.31 |
| Leaf P | -> | Herbivory rate | -0.17 | -0.17 | 0.09 | -1.81 | 0.07 |
| **EM** | **->** | **RDA axis 1** | **-0.33** | **-0.74** | **0.18** | **-4.02** | ******* |
| EM | -> | Herbivory rate | -0.08 | -0.17 | 0.21 | -0.81 | 0.42 |
| EM | -> | Tree biomass | -0.14 | -0.31 | 0.20 | -1.53 | 0.13 |
| **AM** | **->** | **RDA axis 1** | **-0.34** | **-0.77** | **0.21** | **-3.63** | ******* |
| AM | -> | RDA axis 2 | 0.15 | 0.33 | 0.20 | 1.66 | 0.10 |
| **AM** | **->** | **Herbivory rate** | **0.30** | **0.67** | **0.22** | **3.07** | **< 0.01** |
| Tree species richness | -> | RDA axis 1 | -0.05 | -0.13 | 0.23 | -0.59 | 0.56 |
| **Tree species richness** | **->** | **RDA axis 2** | **0.21** | **0.55** | **0.23** | **2.34** | **0.02** |
| Tree biomass | -> | Pathogen infestation rate | 0.16 | 0.16 | 0.09 | 1.70 | 0.09 |
| Tree biomass | -> | Herbivory rate | -0.06 | -0.06 | 0.10 | -0.60 | 0.55 |
|  |  |  |  |  |  |  |  |
| **AM** | **<->** | **EM** | **-0.37** | **-0.07** | **0.02** | **-3.61** | ******* |
| **Tree biomass** | **<->** | **Leaf N** | **0.26** | **0.25** | **0.09** | **2.69** | **< 0.01** |
| **Tree biomass** | **<->** | **Leaf C** | **0.29** | **0.27** | **0.09** | **2.88** | **< 0.01** |
| **Leaf P** | **<->** | **Leaf C** | **0.25** | **0.24** | **0.09** | **2.61** | **0.01** |
| **Leaf P** | **<->** | **Tree biomass** | **0.23** | **0.21** | **0.09** | **2.32** | **0.02** |
| **Leaf C** | **<->** | **Leaf N** | **0.27** | **0.24** | **0.09** | **2.64** | **0.01** |
| Herbivory rate | <-> | Pathogen infestation rate | -0.17 | -0.15 | 0.09 | -1.68 | 0.09 |
| Tree biomass | <-> | RDA axis 2 | -0.09 | -0.08 | 0.07 | -1.12 | 0.26 |
| RDA axis 1 | <-> | Pathogen infestation rate | -0.19 | -0.16 | 0.18 | -0.94 | 0.35 |
| RDA axis 1 | <-> | Herbivory rate | -0.03 | -0.02 | 0.08 | -0.30 | 0.76 |
| RDA axis 2 | <-> | Herbivory rate | -0.10 | -0.09 | 0.09 | -1.06 | 0.29 |
| RDA axis 2 | <-> | Pathogen infestation rate | -0.40 | -0.40 | 0.32 | -1.25 | 0.21 |
| Leaf P | <-> | RDA axis 2 | -0.08 | -0.08 | 0.08 | -1.05 | 0.29 |
| **RDA axis 1** | **<->** | **RDA axis 2** | **-0.53** | **-0.48** | **0.10** | **-4.90** | ******* |
